# Supplementary figures and images for: Artificially inserted strong promoter containing multiple G-quadruplexes induces long-range chromatin modification
Source: eLife. 2024 Aug 19;13:RP96216. doi: 10.7554/eLife.96216 (PMC11333042; doi:10.7554/eLife.96216)

Uncropped image of Figure 1B with relevant samples and bands labelled

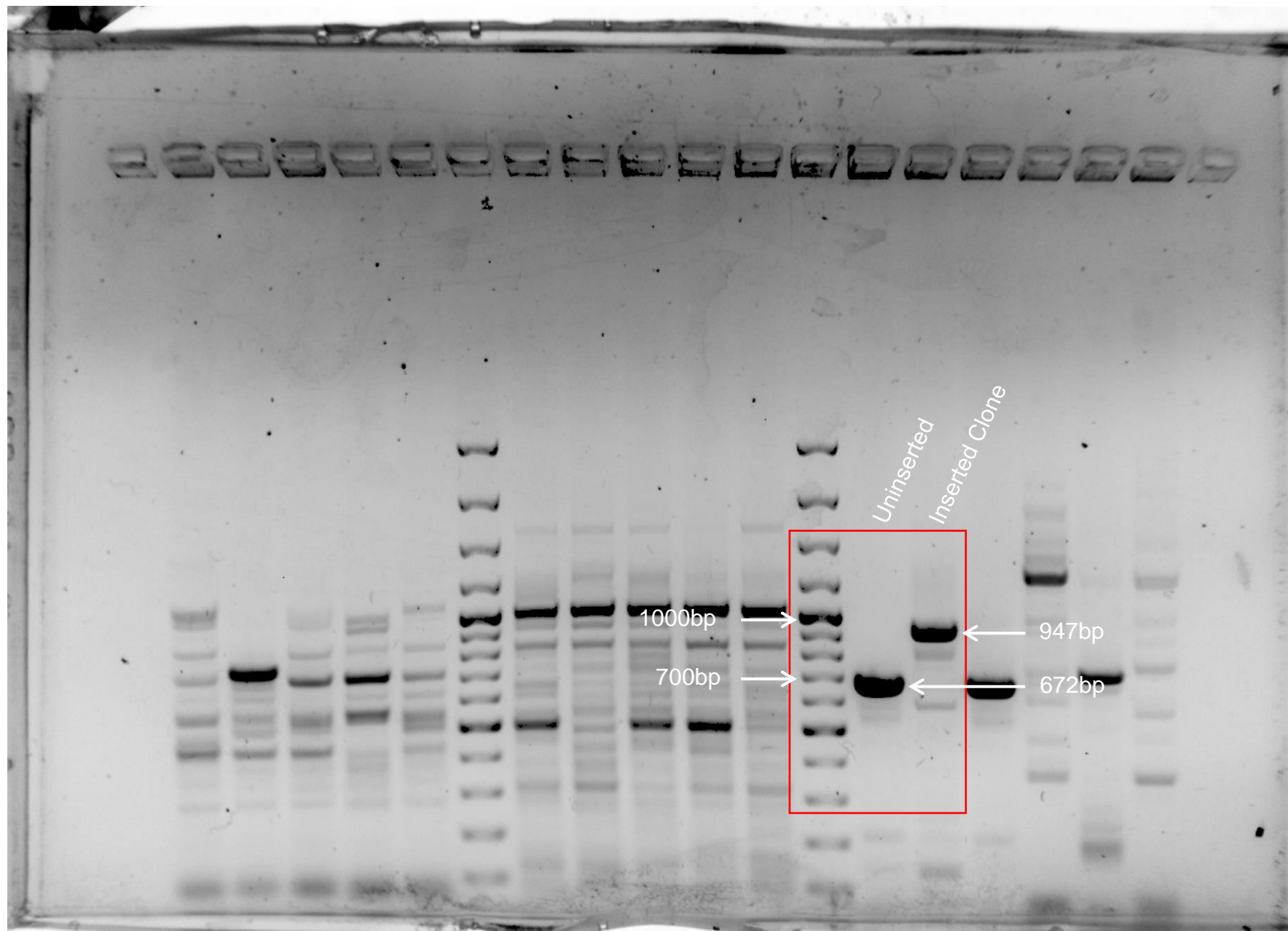

Supplement: Figure 1—source data 1. [file elife-96216-fig1-data1.zip › Figure1SourceData1/Uncropped image of Figure 1B with relevant samples and bands labelled.pdf]

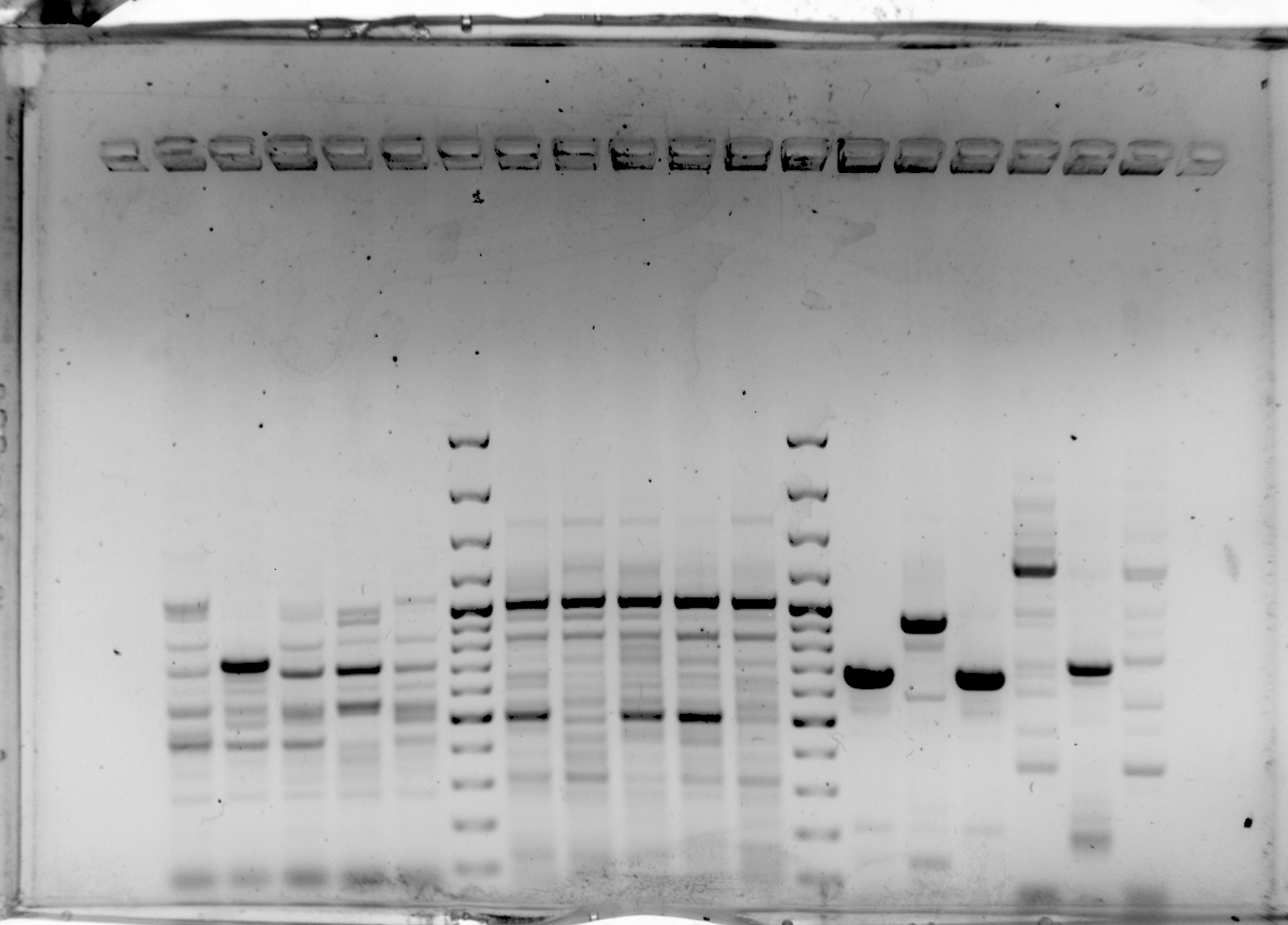

Supplement: Figure 1—source data 1. [file elife-96216-fig1-data1.zip › Figure1SourceData1/Figure 1B uncropped.jpg]

Uncropped image of Figure 7B with relevant samples and bands labelled

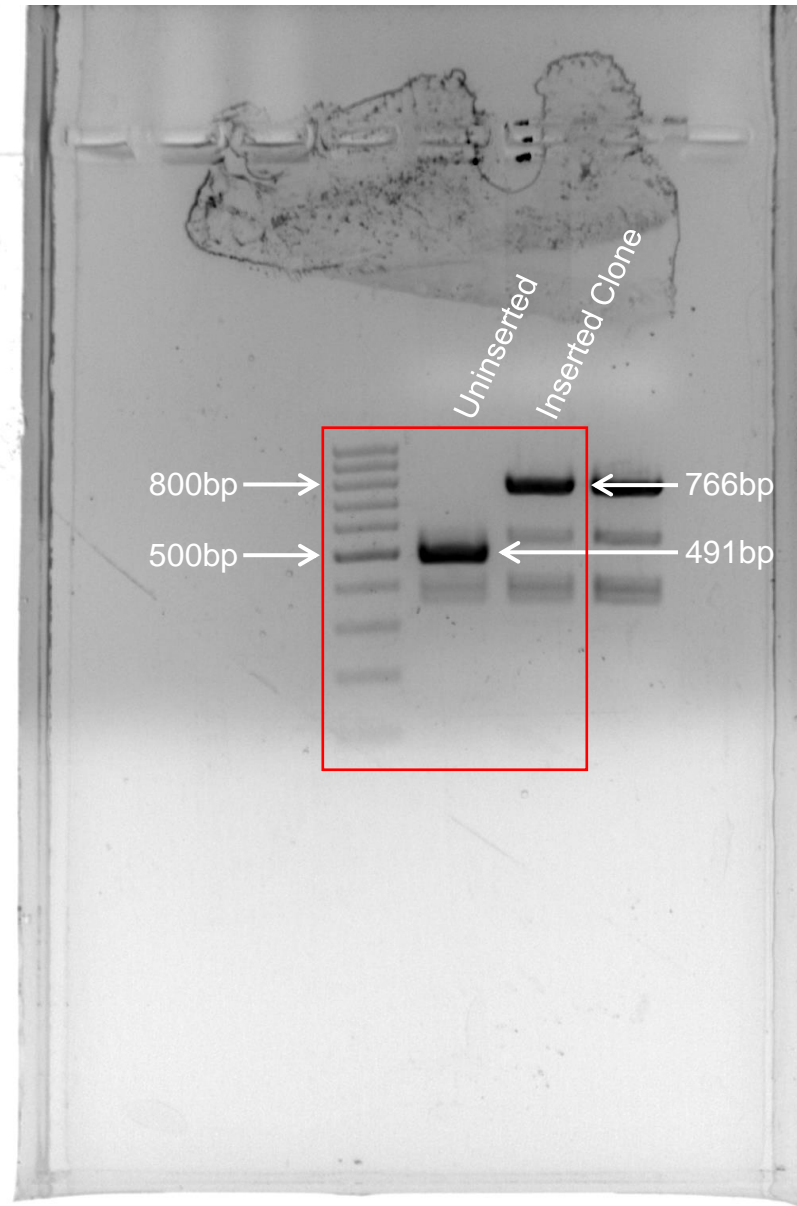

Supplement: Figure 7—source data 2. [file elife-96216-fig7-data2.zip › Figure7SourceData2/Uncropped image of Figure 7B with relevant samples and bands labelled.pdf]

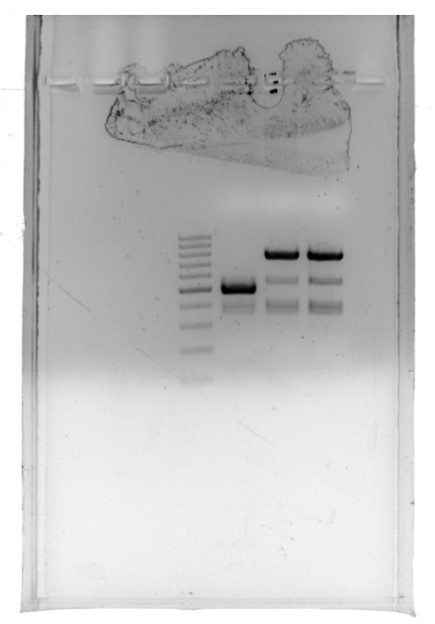

Supplement: Figure 7—source data 2. [file elife-96216-fig7-data2.zip › Figure7SourceData2/Figure 7B uncropped.jpg]
