# Supplementary material for: Artificially inserted strong promoter containing multiple G-quadruplexes induces long-range chromatin modification
Source: eLife. 2024 Aug 19;13:RP96216. doi: 10.7554/eLife.96216 (PMC11333042; doi:10.7554/eLife.96216)
Supplement: Supplementary file 1. — Table showing the number of genome-wide raw Hi-C contacts, actual number of contacts originating from the ±10 kb window with the insertion site at the middle and the comparative analysis (mean, standard deviation and z-score) of these number of contacts with contacts across 10,000 random 20 kb windows across the genome across the 3 samples. [file elife-96216-supp1.docx]

|  | G4-array insert | G4-mutated insert (control) | HEK293T |
| --- | --- | --- | --- |
| Total Hi-C contacts | 649,455,101 | 692,798,603 | 366,957,555 |
| Normalized contacts from insertion site | 6390 | 3133 | 3968 |
| Actual contacts from insertion site (±10 kb, i.e. 20kb window) | 5990 | 3133 | 2102 |
| Mean Hi-C contacts across 10,000 random 20kb windows across the genome | 8910 | 9490 | 5021 |
| Standard deviation of Hi-C contacts across 10,000 random 20kb windows across the genome | 4078 | 4716 | 2806 |
| Z-score for the actual contacts from the insertion site | -0.71 | -1.34 | -1.04 |

**Supplementary File 1**
